# The psychological impact of COVID-19 quarantine on children, and the role of parental support and physical environment design

**DOI:** 10.1007/s44202-021-00002-6

**Published:** 2021-09-28

**Authors:** Mais M. Aljunaidy, Mohamad Nadim Adi

**Affiliations:** Bilkent University, Ankara, Turkey

**Keywords:** COVID-19, Environmental design, Children’s mental health, Child anxiety, Parental stress and support

## Abstract

Coronavirus disease 2019 is a contagious infection that caused a global lockdown and affected children who needed to stay home. There is a lack of knowledge about the role of parental stress and physical environment design on children’s mental wellbeing in quarantine. We hypothesis that COVID-19 quarantine affected child mental health, and that paternal stress or support, and child physical environment including household space, colors, sunlight exposure, and natural views, impacted child mental wellbeing in the quarantine. To assess the effect of quarantine on a child’s mental health, an online survey was administered globally through scientific organizations and social media. Those over 18 years old, and guardians of children were asked to participate in the survey. The survey was filled by 114 guardians from 31 countries. Descriptive statistics were used to summarize the data. Most participants experienced stress in the quarantine and reported child anxiety symptoms including focus reduction, sleeping difficulties, and appetite changes. Family fun activities and encouraging words, were mostly successful in reducing child anxiety. Reporting anxiety symptoms in children were more common in parents who had mental hardships compared to those who did not experience mental problems or had an improved mental status. Physical environment assessment showed that households with bright walls associated with fewer reports of child mental problems compared to households with neutral wall colors, and that most guardians thought that their children’s living space was not sufficient to play and study. Architects can provide evidence-based recommendations for customers to support children’s mental health.

## Introduction

The coronavirus disease 2019 (COVID-19) is a contagious infection caused by the severe acute respiratory syndrome coronavirus 2 (SARS-CoV-2) [1]. The first case of COVID-19 was identified in December 2019, which quickly spread worldwide negatively affecting millions of people around the globe [2]. The infection spreads through droplets that are formed when an infected person coughs or sneezes [3]. COVID-19 infection, which primarily affects the respiratory tract, can spread easily and be deadly [4]. As a countermeasure, people around the globe were instructed to avoid face-to-face socializing, wear face masks, keep social distancing, and in most countries, a curfew was announced. In the curfew, all people had to stay home for months in quarantine with generally only two exceptions of either having an emergency or a job that requires face-to-face interaction. Businesses, learning, and public common areas were all closed in the quarantine period. Among the spaces that were closed in the pandemic were schools. Distance learning was established using classes broadcasted through television programs or online platforms. Therefore, millions of school children around the world needed to stay home for months due to the sudden and unexpected closure of schools worldwide. The fear of COVID-19 infection, isolation from colleagues and friends, changes in the daily routines, and guardians losing their jobs or working from home would have considerably caused stress to children and led to a significant impact on their mental health [5–10]. A study in Hubei province in China showed a 4.7–10.3% prevalence of behavioral problems in school-aged children due to the COVID-19 home quarantine [8]. Those behavioral problems included conduct problems, peer problems, hyperactivity-inattention, and emotional problems [8]. Furthermore, a cross-sectional study in Bangladesh showed that COVID-19 quarantine caused depression, anxiety, and sleeping disorders in children aged between 5 and 15-years-old [10]. In a national survey in the United States, 14% of parents who participated in the study reported worsening emotional health of their children [9].

In the quarantine, parents with young children experienced stress due to the need of balancing work with childcare/home-schooling and due to financial instability [7]. For example, In a study performed in Canada using a questionnaire targeted to parents with young children, about 19% of mothers and 14% of fathers stated that they experienced financial stress due to the quarantine [7]. In the same study, 10% of mothers and 5% of fathers reported concerns about food security [7]. Parental stress can negatively reflect on a child’s mental health. For example, a study in the United States showed that when parents were stressed they had a higher chance of becoming physically abusive to their children than when they were relaxed [5]. Another study in the United States used data collected from reports to Indiana Child Protective Services (CPS), showed that children in areas that stayed home more in quarantine were more likely to be reported as and confirmed victims of child neglect compared to areas with less quarantine restricting hours [6].

Physical environment characteristics including space, wall colors, natural views, and sunlight exposure also have an impact on a child’s mental health. For example, in India, home density (the ratio of the number of occupants to the residential area) was positively correlated with social withdrawal and lower academic achievements in children aged between 10- and 12-years-old [11]. In Spain, a cross-sectional study performed on children (7–10 years of age) found that time spent in green spaces had an inverse correlation with peer relationship problems and hyperactivity/inattention [12]. In the same study, blue spaces (beaches) had a positive impact on the child-peer relationship [12]. In the Netherlands, green space surroundings in people aged between 12- and 65-years-old reduced propensity to psychiatric morbidity [13]. Furthermore, in the United Kingdom, natural green spaces reduced emotional problems in children aged 3-years-old, 5-years-old, and 7-years-old [14]. Furthermore, sunlight exposure is essential for child development and mental health [15]. Sunlight exposure is essential for vitamin D production in the human body, and vitamin D deficiency can be associated with many abnormal neuropsychiatric outcomes such as depression, schizophrenia, and autism [15]. However, the possible role of the physical environment in supporting child mental health in the COVID-19 quarantine is still unknown.

Studies assessing child psychology due to the COVID-19 pandemic were usually mostly focused on limited regions or were performed on a national level [5–10]. Therefore, a global survey was needed to show a bigger picture of how COVID-19 quarantine affected child mental health around the world. Furthermore, no study assessed the role of the physical environment and how this might impact a child’s coping with the quarantine. We hypothesize that COVID-19 quarantine has a negative impact on a child’s mental health, and that parental factors including feeling stressed or being supportive play a role in increasing or reducing child mental problems. Furthermore, a child’s physical environment including household space, colors, views, and sunlight exposure may play a supportive role in improving a child’s mental wellbeing in quarantine.

## Methods

### Ethical approval

Ethical approval was obtained from Bilkent University, and followed the principles endorsed by relevant professional bodies in particular to the Declaration of Helsinki (WMA), Ethical Principles of Psychologists and Code of Conduct (APA), and Ethical Standards for Research with Children (SCRD).

### Data collection

A team of cross-disciplinary researchers experienced in psychology and in architecture used a survey that included 32 questions related to child psychology and physical environment design. The survey was adapted from previously published surveys related to environmental design and child/parent’s mental health [5, 7, 8, 12, 16], and was uploaded online using the “Google Forms” website. A link to the survey was then distributed through the email lists of academic institutions and scientific organizations (please, see the acknowledgement section for details), and through social media platforms (Facebook and WhatsApp). The survey was available in 2020 from mid-June until the end of August when schools were over, and many countries started at that time to ease COVID-19 quarantine restrictions. The timing was chosen so parents can still remember their child’s experience studying from home away from any face-to-face interaction with teachers, friends, and colleagues. participants received a clear and simple written explanation of the study that was available for them to read before starting the survey. Furthermore, the message associated with the survey link asked only those who were over 18 years old and were guardians of children to participate in the study. The message indicated that participation in the survey would be confidential, the responses would be anonymized, and that the participants could withdraw from the survey at any time without giving justification. A guardian/parent of a child was defined as a person who was legally responsible for caring of a minor child regardless of being a male or a female.

The survey was distributed globally in the English language. The survey was divided into two major sections. The first section was related to the guardians’ status in the quarantine, and included the participants’ demographic information, employment status in the quarantine, financial status in the quarantine, and changes in the guardian’s responsibility regarding caring for their children in the quarantine. The second major section in the survey was written to ask about a child’s psychological status due to the quarantine, and possible supportive factors that could improve a child’s mental health such as parental support and physical environment. This second section included a child’s knowledge about the pandemic, a child’s psychological status in the quarantine as reported by the guardians, and a child’s physical environment and its possible relation with the child’s mental wellbeing. In the survey questions, the focus was mainly on school children as children at a younger age were less aware of the pandemic than the children at school age [17]. Descriptive statistics were analyzed and organized in charts using the “Google Forms” website and Microsoft Excel Worksheet.

## Results

### Participant demographic information

The survey was filled by 114 participants (guardians) from 31 countries (Table 1). Among people who took part in the survey, 2 participants chose not to mention what country they lived in in the quarantine. The majority (92.10%) of the respondents were between the age of 30 and 49 years; another (3.50%) were between 50 and 64 years, (2.60%) between 18 and 29 years, and (1.80%) were above 65-years-old. More than two-thirds (65.80%) of the respondents were females; 33.30% were males, and 0.90% were nonbinary. Most of the participants (92.00%) were married; 4.50% were divorced, and the rest (3.60%) preferred not to say.

**Table 1:** Percentage of the survey’s participation based on the countries

Countries	Percentage of participation (%)
Canada	25.89
Turkey, Spain, and Jordan	26.79 (8.93 each)
United Kingdom	6.25
United States of America	5.36
Germany	4.46
Czech Republic	3.57
Italy	2.68
Kingdom of Saudi Arabia, Poland, and Bosnia	5.37 (1.79 each)
Serbia, Greece, Cuba, Sweden, Yemen, Syria, United Arab Emirates, Portugal, Indonesia, Morocco, Qatar, Norway, Nigeria, Ireland, Peru, Lithuania, Austria, France, and Belgium	16.91 (0.89 each)

### Participant employment status in the quarantine

**Fig. 1:**
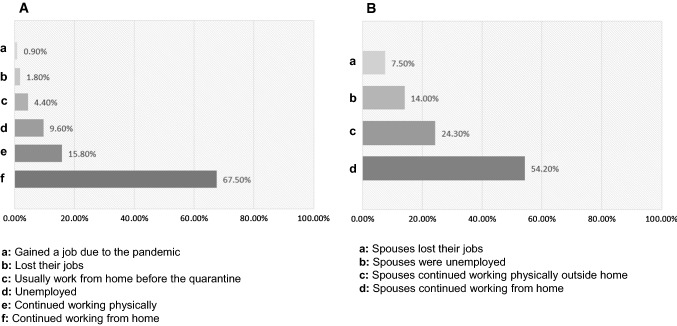
Employment status in the quarantine for both the study participants and their spouses. A: Most participants continued working from home instead of going physically to their working places (**A**), and over half of the participants had their spouses working from home too (**B**)

In the pandemic quarantine, most parents needed to continue working from home instead of going physically to their workplaces (Fig. 1A). Most of the participants (87.00%) were working full-time throughout the quarantine period and the rest were part-timers (13.00%). Just over half of the participants had a spouse who continued working from home (Fig. 1B). Around a quarter of the participants reported that their spouses continued working physically outside the home (Fig. 1B). The rest of the participants indicated that their spouses were either originally unemployed or lost their jobs due to the pandemic (Fig. 1B). When the spouses continued working throughout the quarantine period, the majority (81.10%) of the spouses were full-timers.

### Participant financial status in the quarantine

**Fig. 2:**
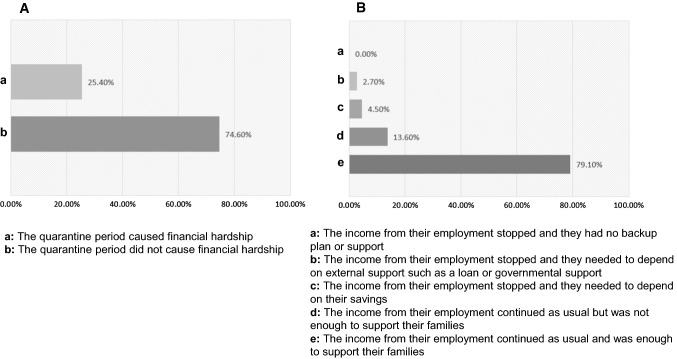
Participant financial status in the quarantine. Over a quarter of the study participants reported that the quarantine period caused financial hardship on them (**A**), but no one indicated that their income stopped or that they had no backup plan (**B**)

Over a quarter of the study participants reported that the quarantine period caused financial hardship on them (Fig. 2A), but no one indicated that their income was abolished completely or that they had no backup plans (Fig. 2B).

### Changes in the guardians’ responsibilities regarding caring for their children in the quarantine

**Fig. 3:**
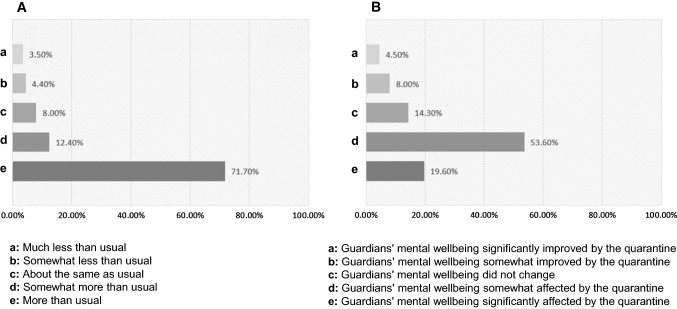
Time committed to taking care of children and parental mental health. Most guardians needed to take care of their children more than they usually did before the quarantine (**A**). The quarantine caused either severe mental stress, or somewhat affected the mental wellbeing in most of the guardians (**B**)

46.00% of the participants had at least two children, and 34.50% of the participants indicated that they had only one child. 72.30% of the participants had children of school age. Due to the quarantine, most guardians said that they needed to take care of their children more than they usually did before the quarantine (Fig. 3A). The quarantine caused either severe mental stress or somewhat affected the self-reported mental wellbeing of the guardians (Fig. 3B). 46.91% of those guardians who had mental distress reported anxiety symptoms in their children. Over a quarter of the participants reported no changes in their mental wellbeing or improvement in their mental health due to the quarantine (Fig. 3B). Only 30.00% of those guardians who had no changes or improvement in their mental wellbeing reported anxiety symptoms in their children.

### Child’s knowledge about the pandemic

Most of the children were informed about the symptoms, and how to protect themselves from COVID-19 (74.10% and 90.20% respectively). About two-thirds (63.20%) of the parents searched online and applied ways to promote handwashing at home.

### Child psychological status in the quarantine as reported by their guardians

**Fig. 4:**
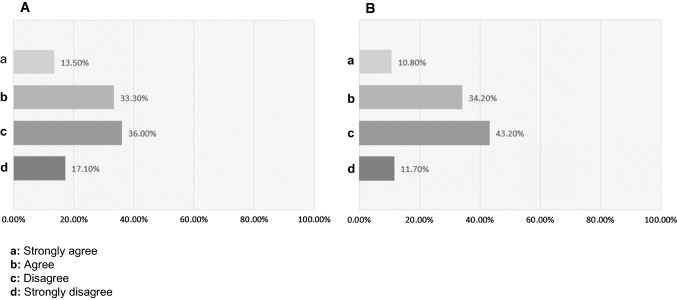
Child psychological status in the quarantine as reported by their guardians. **A** Guardians’ thoughts about whether their children were mentally and emotionally prepared for the possibility of continuing school learning online in the following year (2021). **B** Guardians’ thoughts whether their children were mentally and emotionally prepared for the possibility that they might need to wear masks outside their homes for the next 2 years

42.90% of the study participants reported that COVID-19 caused anxiety to their children, while 57.10% reported no child anxiety symptoms. Over half of the guardians thought that their children were not mentally and emotionally prepared for the possibility of continuing school learning online in the following year (2021), (Fig. 4A). More than half of the guardians thought that their children were not mentally and emotionally prepared for the possibility that they might need to wear masks outside their homes for the next 2 years (Fig. 4B). The guardians who reported that their children were not adjusting to the usage of masks claimed that the main reasons were because their children were too young to understand the concept of wearing a mask (45.20%), the children think that the mask is not comfortable (35.70%), or they needed longer time to adapt (26.20%).

Over half (57.10%) of the guardians who reported that their children were anxious due to the pandemic, claimed that their children also showed new and unusual behaviors such as a reduction in their abilities to focus on their studies (56.00%), sleeping difficulties (46.00%), changes in their appetite (34.00%), Being on edge (10.00%), or self-harm (4.00%). Other pathopsychological symptoms did not affect more than 2.00% of the children who were reported by their guardians to have psychological problems due to the pandemic. Those pathopsychological problems included phobias, refusing to leave their rooms, feeling extremely bored due to no friend interactions, increased dependence on parents, and hyperactivity.

More than half of the study participants (52.20%) tried to reduce their child’s anxiety to the pandemic by searching for and applying some supportive methods including fun activities and encouraging words. Guardians reported that those supportive methods were effective and successful in 67.60% of the cases.

### Child physical environment and its possible impact on child mental health

Most of the participants (85.71%) reported having neutral wall colors in their households (beige, white, or light brown), and 43.75% of those who had neutral wall colors claimed that their children had mental problems in the quarantine. However, for those who reported having bright colors walls (14.71% of the participants) only 37.5% of them claimed that their children had mental problems in the quarantine.

**Fig. 5:**
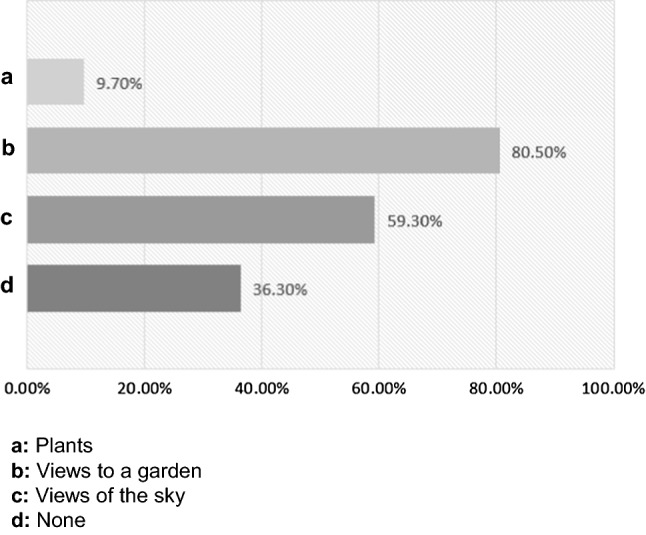
Child’s blue and green space surroundings. Most of the participants had views of the sky in their households, but only about a third of them had plants, and about half of them had a garden view

Most guardians (97.30%) claimed that their living spaces got enough natural light during the day. Only 2.70% of the participants said that their homes did not have access to sunlight. About half of the participants had garden views, about a third of them had plants, and most of them had views of the sky in their households (Fig. 5).

**Fig. 6:**
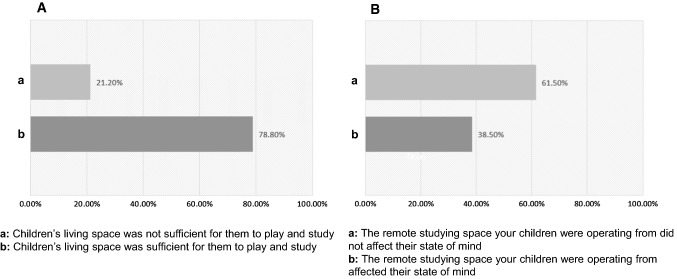
Child’s living space. Most guardians claimed that their children’s living space is not sufficient to play and study (**A**), and that the remote studying space their children were operating from affected their child’s state of mind (**B**)

Many guardians thought that their child’s living space was not sufficient to play and study (Fig. 6A), and that the remote studying space their children were operating from affected their child’s state of mind (Fig. 6B). Those who reported child’s living space insufficiency indicated that their child’s living space led to a reduction in the child’s ability to study (57.40%), caused difficulties to sleep (25.50%), and changed the appetite of the child (14.90%). Guardians were asked about what aspects of their environment they thought they would change to improve their child’s performance and mental health in the quarantine. Those guardians were able to check “anything that applies” of the following options: wall colors, natural light, natural views, or more spacious space. The results showed that 57.00% of the participants chose to have more living space, 27.40% of the participants chose to have more access to natural elements such as garden or sky views, 23.40% of the participants proposed a better room sunlight exposure, and 6.4% of the participants chose a possible different wall color.

## Discussion

Our survey showed that most of the participants and their spouses continued working full-time from home in quarantine, and over a quarter of those participants experienced financial hardships. Most participants had children at school age; and expressed severe mental distress due to the need to balance work responsibilities and taking care of children at home, and due to the financial instability caused by the quarantine. Parental distress due to COVID-19 might be a risk factor for the anxiety children experienced in the pandemic. Major symptoms of child anxiety were shown as a reduction in their ability to focus on their studies, sleeping difficulties, and changes in their appetite. More than half of the study participants tried to reduce their child anxiety in the COVID-19 pandemic by searching and applying some supportive methods including fun activities and encouraging words. In most cases, those supportive methods were effective and successful in reducing child anxiety. The assessment of the physical environment’s role on a child’s mental health in the quarantine period showed that many guardians thought that their child’s living space was not adequate nor ideal to play and study. Furthermore, the report of child mental problems was higher in the households with neutral wall colors compared to those households with bright wall colors, suggesting that designers should encourage their customers to have brighter walls in homes with little children.

Parental distress can have a significant impact on a child’s mental health [5, 18]. For example, a survey in the United States showed that adult anxiety due to COVID-19 led to a negative impact on guardians’ behavior towards their children which included being psychologically and physically abusive [18]. Another survey in the United States found that when parents were stressed they had a higher chance of becoming physically abusive to their children than when they were relaxed [5]. These findings agree with our study, as we suggest that parental distress due to the difficulty of time management and the increased responsibilities in the pandemic can be one of the risk factors that might impact a child’s mental health. Therefore, introducing shorter working hours for parents and shorter studying hours for children might ease the pandemic hardship on families, and improve both a guardian’s and a child’s mental health.

Family fun activities such as arts and crafts, watching a family movie, or physical activities are essential for child development and mental health [19–21]. Our study showed that about half of the guardians searched and applied methods to reduce child anxiety and worries in quarantine, and when guardians were asked if they think that family activities at home reduced their children’s anxiety in COVID-19, most of them agreed. Our results about the usefulness of family activities in improving child mental health were supported by other studies. For example, in Canada, self-report data collected from vulnerable children with a mean age of 10.34 years showed that arts-based mindfulness group programs improved child mood, emotion, coping, and ability to pay attention and focus [19]. Furthermore, physical activities can reduce anxiety and depression in children. For example, a survey-based study in the United States showed that physical activity ≥ 60 min on 1–3 days/week or 4–6 days/week in children aged between 6- and 17-years-old associated with a significantly lower level of anxiety and depression compared to children who had 0 days/week of physical activity [21]. Reading storybooks is an established helping technique that is widely used within a variety of counseling or therapeutic modes, and enables a young person to identify with a fairy tale or to create their own version up to unlock feelings of guilt, sadness, or mistrust [20]. Therefore, family fun activities can be another supportive method that can be used to reduce child anxiety in quarantine. However, what types of family activities exactly are the most useful in supporting child mental health in the quarantine still needs further investigations.

The effect of environmental design on child mental health and emotions has already been established [11–14]. However, to the best of our knowledge, our study was the first to address the link between home design and improving child mental health in the COVID-19 pandemic. Physical environment design aspects that can impact child development and mental health were reviewed in Ferguson et al. [22]. In our survey, we included four major features of home interior design that can impact a child’s mental health [11, 14, 15, 23]. These interior design aspects were space (living area where a child is performing most of the daily activities), sunlight exposure, wall colors, and natural views including exposure to green space or sky views. There are other housing features that can impact a child’s mental health including neighborhood quality, housing location, and housing quality (housing assistance, cleanliness, moisture, pests, noise, accessibility, injury risks) [24–26]. However, as families were supposed to be in quarantine, we focused mainly on interior design features. Furthermore, we chose to keep our study focused and generalized. Therefore, assessment of the effect of housing quality, which was outside the scope of our study, was not included in our survey. Our survey showed that most guardians were not satisfied with their child’s living space and indicated that the living space was not sufficient for their children to play and study in the lockdown. Interior designers usually think that children can go outside to play at any time, so commodious places were usually left to the outdoor spaces, while home spaces were left tiny [27]. Choosing to keep home spaces tiny by interior designers could also be attributed to customers’ requests, smaller-sized families, the unavailability of land. The effect of small household spaces on children was not apparent before the pandemic because children could still have access to outdoor activity spaces such as classrooms, parks, and play areas where children can go with their peers or families. The COVID-19 pandemic showed that going and playing outside might not always be an option, and home design should take into account that children should have a reasonable space at home to run and play.

The colors of the surrounding environment are an important aspect of environmental design [28]. Color preference is different and depends on age; while adolescents prefer neutral colors, younger children prefer brighter colors [29]. Therefore, most children’s play areas are usually designed with strong colors (blue, red, green, yellow). It is established that colors in the surrounding environment can affect energy levels, mood, and mental clarity [30]. A previous study showed that wall color can impact children’s cooperative behavior [23]. Furthermore, cool colors permit concentration, and wall colors in the classroom affect productivity and accuracy [29]. We, therefore, suggested that the lack of access to colorful spaces might negatively impact a child’s mental health in quarantine. Our results showed that in most homes the wall color scheme was neutral (white, beige, gray, or light brown). The report of child mental problems in the quarantine was higher in those who had neutral wall colors compared to those who had bright wall colors, suggesting that designers should encourage brighter walls in homes with little children.

Sunlight exposure is essential for children’s development and mental health [15]. Sunlight exposure contributes to the production of vitamin D in human body, and vitamin D deficiency can be associated with many abnormal neuropsychiatric outcomes such as depression, schizophrenia, and autism [15]. Daily exposure of 30 min is adequate for the body to produce enough amount of vitamin D [31]. In our study, most guardians indicated that their households get adequate sunlight. Therefore, lack of sunlight exposure was not a factor that played a role in child mental problems in quarantine. However, about a quarter of the study participants proposed that better room sunlight exposure would possibly cheer up their children and improve their performance and mental health in quarantine.

Some limitations of our study were that the color of furniture was not taken into consideration when the environmental design was assessed as we were looking at the general color scheme of the rooms rather than specific parts of these rooms. The survey was conducted in the English language only. The distribution of the survey in English might have limited the number of participants in the study. However, getting responses in all possible languages was challenging as it would necessitate a significant number of native speaker interpreters for each language. Otherwise, the accuracy of the answer’s meaning might change due to poor translation. Measures were not all the same. This study facilities bigger studies with more collaborations.

## Conclusion

We conclude that the COVID-19 lockdown caused significant distress to the guardians and mental issues for their children. Attempts by the guardians to reduce the anxiety of their children through family activities and encouraging words were mostly successful as reported by the guardians, emphasizing the fact that guardians’ support was helpful in supporting children’s mental health in quarantine. Furthermore, the pandemic and the subsequent lockdown highlighted the fundamental role of environmental design in supporting children’s mental wellbeing. Although customers and available resources are still major factors in determining many factors in housing design, architects and interior designers can provide evidence-based recommendations about planning wider living room spaces and recommend brighter wall colors to support the mental health of children.

## Data Availability

The datasets generated and analyzed in the current study are not publicly available due to the fact that participants in this study were explicitly informed that their data will only be used for the purposes of this study but are available from the corresponding author on reasonable request.
